# Evaluating antimicrobial prescribing in a Tertiary Healthcare Institution in Nigeria

**DOI:** 10.1186/s40545-021-00380-1

**Published:** 2021-11-30

**Authors:** Angus Nnamdi Oli, Nwanneka Onyeaso, Stephen Chijioke Emencheta, Chijioke M. Ofomata, James-Paul Kretchy, Augustine Okhamafe, Grace N. Ele

**Affiliations:** 1 Department of Pharmaceutical Microbiology and Biotechnology, Faculty of Pharmaceutical Sciences, Nnamdi Azikiwe University, Agulu, Nigeria; 2 Department of Clinical Pharmacy and Pharmacy Practice, Faculty of Pharmacy, University of Benin, Benin City, Nigeria; 3 Department of Pharmacy, Nnamdi Azikiwe University Teaching Hospital, Nnewi, Nigeria; 4 Department of Pharmaceutical Microbiology and Biotechnology, Faculty of Pharmaceutical Sciences, University of Nigeria, Nsukka, 410001 Nigeria; 5 Department of Clinical Pharmacy and Pharmacy Management, Faculty of Pharmaceutical Sciences, Agulu Nnamdi Azikiwe University, P.M.B 5025, Awka, Nigeria; 6 Department of Physician Assistantship Studies, School of Medicine and Health Sciences, Central University, P. O. Box DS 2305, Accra, Ghana; 7 Department of Pharmaceutics, Faculty of Pharmacy, University of Benin, Benin City, Nigeria

**Keywords:** Pharmacy practice, Antimicrobial use, Rational prescribing, Essential drug list, Drug utilization

## Abstract

**Background:**

Regular evaluation of antimicrobials prescriptions is important for optimal use.

**Objective:**

This study determined the prescription patterns, class and costs of antimicrobials in the adult out-patient pharmacy of a Teaching Hospital in Nigeria.

**Methods:**

A 1-year retrospective study from 1st January to 31st December 2018. The data, which included identification code, age, sex, antibiotics prescribed, number of antibiotics per prescription, number of medicines per prescription, dosage form, generic prescribing, drug on the essential drug list, and cost, were used in the analysis. The Chi-square test and Analysis of Variance were used to compare our data with the WHO–developed antimicrobial prescription Guidelines for Anatomical Therapeutic Chemical and Defined Daily Dose assignment of 2019.

**Results:**

From 450 patient records, significantly more females (70%) were prescribed with antimicrobials (*P* = 0.0038). The prescription pattern showed that antimicrobials selection by class was significantly different (*P* < 0.0001) (top three being Amino-penicillin > Nitroimidazoles > Fluoroquinolone). In addition, age differed significantly (*P* < 0.0001) with 46–50 as the highest class. Dosage forms profile showed that the percentage of encounter with injections prescribed (1.8%) was less than WHO recommendation (13.4–24.1%). Most of the prescriptions (84.22%) were from the Essential Drug List. The average cost of prescriptions with two antimicrobials was the highest ($14.0807), then three ($10.7949), and one ($6.39858). The average number of drugs per prescription that had one (4.28), two (4.46), and three (5.55) antimicrobials, respectively, were more than double the average (2) recommended by WHO.

**Conclusion:**

The study showed that most of the patterns are within limit, however, highlights the need for frequent evaluation.

## Background

The challenges of irrational prescription of antimicrobial drugs, such as antibiotics, are creating enormous public health problems globally and in developing countries in particular [1–3]. Antibiotics are medications that can destroy or inhibit the growth of bacteria by either selectively killing or inhibiting the development of disease-causing bacteria [4]. They play a pivotal role in combating disease and maintaining health especially in developing countries, where infectious diseases are still a big challenge [4]. The World Health Organization (WHO) stated that more than 50% of all drugs are irrationally prescribed, with over 50% of patients having problems with adherence to the prescribed drugs [5]. The improper utilization of these antibiotics is often caused by medical practitioners or pharmacists who prescribe antibiotics for health problems that do not require antibiotics for treatment [6].

Regular and timely evaluation of antibiotic utilization and continuous epidemiological monitoring [7] are some interventions used to mitigate the associated problems with irrational use, prevent the development of antibacterial resistance and reduce the spread of bacterial infections [7]. Antimicrobial drug utilization which has been defined as the marketing, distribution, prescription and use of drugs in a society, has medical and social consequences, and if not properly utilized may aggravate the health outcome of persons with infectious diseases [8]. Some of the benefits of proper healthcare delivery systems in a country include the safety and effectiveness of prescribed drugs and their affordability by the average person. Inappropriate and overuse of antibiotics are examples of common types of irrational use which could lead to poor treatment outcomes, drug resistance and high economic burden on patients [9]. Antibiotic resistance is a global health crisis and is one of the greatest challenges for public health and affects both developing and developed countries [4]. The problems associated with the irrational use of antibiotics in developing countries, such as Nigeria, may be worsened by limited funding by governments to procure enough quality medications and the lack of safe-guarding against the peddling of fake and sub-standard antibiotics across the borders [9, 10].

Previous studies [11, 12] have evaluated the use of antibiotics in children visiting the out-patient pharmacy and among geriatric in-patients, but not in adults visiting the adult out-patient pharmacy. This information would add to the knowledge and understanding of the antibiotics utilization among the adult population visiting the out-patient pharmacy of a tertiary health institution in Nigeria. The study outcome can also inform the development of standards for guidelines for antimicrobial use in teaching hospitals in Africa. Thus, our study, therefore, sought to evaluate the demographic characteristics of patients in the adult out-patient pharmacy of a Teaching Hospital in Nigeria, describe the pattern antimicrobial utilization, determine the antibiotics prescriptions per encounter, find out the availability of the prescribed antibiotics on the essential drug lists and determine the cost associated with antimicrobial use.

## Materials and methods

### Study area

This was a retrospective study using prescriptions from the adult out-patient pharmacy of a Teaching Hospital in Nigeria, from 1st January to 31st December 2018. The Teaching Hospital is a federal government-owned referral hospital. The 350-bed hospital offers medical care to patients in the city of location and its surrounding towns. It is also a training centre for medical students and doctors as well as other healthcare practitioners. The adult out-patient pharmacy serves the adult out-patient clinics in the hospital. The major kinds of prescriptions received in the pharmacy are the National Health Insurance Scheme and Drug Revolving funded prescription.

### Data collection tool

All the prescription sheets containing one or more antibiotics in the year from 1st January to 31st December 2018 were used in the study. The information for data collection included the following; Identification code, age, sex, antibiotics prescribed, number of antibiotics per prescription, number of medicines per prescription, dosage form, generic prescribing (yes/no), Drug on Essential Drug List (yes/no) and Cost of the antibiotic(s).

### Data management and analysis

Data were evaluated using the WHO-developed antibiotic prescription indicators [13] and the guidelines for Anatomical Therapeutic Chemical (ATC) classification and Defined Daily Dose (DDD) assignment of 2019 [14]. The ATC classification system and the DDD as a measuring unit are recommended by the WHO for drug utilization monitoring and research. The WHO indicators calculated included: average number of drugs per encounter, percentage of drugs prescribed from generics, percentage of encounters with an antibiotic prescribed, percentage of encounter with an injection prescribed, and percentage of drugs prescribed from the National Essential Medicine List (EML). The rationality of prescriptions was evaluated using some of the WHO core drug prescribing indicators, that is, (a) the average number of drugs per encounter, (b) percentage of encounters with an antibiotic, (c) percentage of encounters with an injection, and (d) percentage of drugs prescribed from the essential drugs list or formulary. The Chi-square test (to test for the difference in sex distribution and trend analysis of essential drug list compliance of prescribed drugs) and Analysis of Variance (ANOVA) (to test for the difference in the selection of antibiotics according to the class and the difference in the prescribed dosage forms) were used to compare our data with the WHO developed antimicrobial prescription guidelines for ATC and DDD assignment of 2019. GraphPad Prism 5.0 Software was used for the statistical analysis.

### Ethical consideration

The study protocols were approved by the Ethics Committee of Nnamdi Azikiwe University Teaching Hospital, Nnewi. Approval number: NAUTH/CS/66/VOL.12/005/2019/002.

## Results

### Demographic characteristics of participants

The Chi-square analysis of the sex distribution (Table 1) in the prescriptions showed that out of the 450 adult patients prescribed with antimicrobials, the number of females (*n* = 317; 70.44%) were significantly higher (*P* = 0.0038) than males (*n* = 130; 28.89%). Less than 1% (4/450) of the prescription sheets had no specific sex indicated, while 12% (54/450) had no specific age indicated in their prescriptions. In addition, the class distribution of the patients’ prescriptions were significantly different (*P* < 0.05). Patients in the age group of 36–40 years (10%) for males and 46–50 years (17.03%) for females were prescribed maximum antibiotics, while patients in the age group of 81–85 years (0.769%) for males and 86–90 years (0.44%) were prescribed the least amount of antibiotics. The age group, between 46 and 50 years had the highest antimicrobial prescriptions across both male and female groups (13.56%), followed by the 41–45-year group (12.44%).

**Table 1:** Sex distribution of the study participants

Age (years)	Gender			Total
	Male *n* (%)	Female *n* (%)	Sex^a^ *n* (%)	
Adult/no specific age	13 (24.07)	39 (72.22)	2 (3.70)	54
16–20	4 (40.00)	6 (60.00)	***	10
21–25	8 (33.33	16 (66.67)	***	24
26–30	11 (42.31)	15 (57.69)	***	26
31–35	11 28.95)	27 (71.05)	***	38
36–40	13 (23.64)	42 (76.36)	***	55
41–45	12 (21.43)	43 (76.79)	1 (1.79)	56
46–50	7 (11.48)	54 (88.52)	***	61
51–55	12 (27.91)	31 (72.09)	***	43
56–60	12 (50.00)	12 (50.00)	***	24
61–65	11 (52.38)	10 (47.62)	***	21
66–70	5 (35.71)	9 (64.29)	***	14
71–75	6 (37.50)	10 (62.50)	***	16
76–80	2 (50.00)	2 (50.00)	***	4
81–85	1 (50.00)	1 (50.00)	***	2
86–90	2 (100.00)	0 (0)	***	2
Total	130 (28.89%)	317 (70.44%)	3 (1%)	450 (100%)

^a^Sex not indicated on prescription form, ***no values recorded for age groups

### Drugs on essential drug list (EDL)

The Chi-square test of the 450 prescriptions reviewed for trend analysis of essential drug list compliance showed no significant difference (*P* = 0.6935). A greater percentage (*n* = 379; 84.22%) of the prescriptions had all the drugs from the Essential Drug list (Table 2). Twelve percent (*n* = 54; 12%) of the prescriptions had none of the drugs from the essential drug lists, while (17; 3.78%) had a mixture, some of the prescribed drugs were in the essential drug list and some were not.

**Table 2:** Drugs prescribed from essential drug list

Sex	Essential drug list			Total
	Yes	No	Yes/no	
Male	108	18	3	129
Female	266	36	14	316
Nil sex	5	0	0	5
Total	379 (84.22%)	54 (12%)	17 (3.78%)	450 (100%)

Yes = All the drugs in the prescription are from essential drug list; No = All the drugs in the prescription are NOT from essential drug list; Yes/no = some of the drugs in the prescription are from Essential Drug list; Nil Sex = gender not indicated in the prescription

### Antimicrobial selection

The Two-Way ANOVA of Antibiotic Use Profile (Table 3) showed that antibiotic selection by class was significantly different and accounted for 44.02% of the total variance seen in the selection practice/prescription pattern (*P* < 0.0001). Amino-penicillin (230; 37.28%) was the most prescribed class of antibiotic in the year in review, followed by Nitroimidazoles (111; 17.99%) and fluoroquinolone (106; 17.18%), the least prescribed antibiotic was Lincosamides (2; 0.32%). In addition, the age of participants accounted for 19.48% of the total variance seen in the selection practice and so, age significantly affected the selection practice/prescription pattern with a *P* < 0.0001.

**Table 3:** Antibiotic use profiling

Antibiotic class and Number of times prescribed (%)									
Age (years)	Amino-penicillin	Cephalosporin	Nitroimidazoles	Fluoroquinolones	Sulphonamide	Macrolide	Penicillin	Tetracycline	Lincosamides
No specific age	17	8	12	18	0	9	0	8	0
16–20	5	2	2	1	0	3	0	1	0
21–25	13	2	13	3	0	3	0	2	0
26–30	14	8	5	5	1	5	0	2	0
31–35	22	5	12	6	1	4	0	0	0
36–40	32	7	8	13	1	10	0	1	0
41–45	32	5	9	11	1	15	0	1	0
46–50	40	5	12	9	0	17	2	1	0
51–55	20	6	11	11	2	2	1	1	0
56–60	13	1	11	8	0	4	0	1	0
61–65	4	1	6	11	0	4	1	1	0
66–70	4	6	3	5	0	0	0	0	1
71–75	11	1	2	3	0	5	0	0	0
76–80	2	0	2	2	0	0	0	0	1
81–85	0	0	2	0	0	0	0	0	0
86–90	1	0	1	0	0	1	0	0	0
Total (617)	230	57	111	106	6	82	4	19	2

### Dosage form profile

The One-Way ANOVA of dosage forms profile (Table 4) showed that the oral dosage forms (*n* = 420; 97%) were significantly more prescribed (*P* < 0.0001) compared with any other dosage form encountered in the study. There was no significant difference in the prescription patterns of parenteral and other dosage forms (*P* > 0.05).

**Table 4:** Distribution of dosage forms of prescribed antibiotics

Age (years)	Oral *n* (%)	Parenteral *n* (%)	Other forms *n* (%)	Total
AD	54 (100)	0 (0.00)	0 (0.00)	54
16**–**20	10 (90.91)	1 (9.09)	0 (0.00)	11
21**–**25	23 (95.83)	0 (0.00)	1 (4.17)	24
26**–**30	25 (92.59)	1 (3.70)	1 (3.70)	27
31**–**35	38 (97.44)	1 (2.56)	0 (0.00)	39
36**–**40	52 (94.55)	2 (3.64)	1 (1.82)	55
41**–**45	54 (98.18)	0 (0.00)	2 (1.82)	55
46**–**50	61 (98.39)	1 (1.61)	0 (0.00)	62
51**–**55	40 (93.02)	2 (4.65)	1 (2.33)	43
56**–**60	17 (100)	0 (0.00)	0 (0.00)	17
61**–**65	20 (100)	0 (0.00)	0 (0.00)	20
66**–**70	10 (100)	0 (0.00)	0 (0.00)	10
71**–**75	12 (100)	0 (0.00)	0 (0.00)	12
76**–**80	1 (100)	0 (0.00)	0 (0.00)	1
81**–**85	1 (100)	0 (0.00)	0 (0.00)	1
86**–**90	2 (100)	0 (0.00)	0 (0.00)	2
Total	420 (97.00)	8 (1.85)	5 (1.15)	433

*AD* adult patients with no specified age entry

### Cost of antimicrobial prescriptions

The result of the cost analysis of antibiotics prescriptions (Table 5), showed that the average cost of prescriptions with two antibiotics was the highest (14.0807 USD), followed by prescriptions with three antibiotics (10.7949 USD), and then prescriptions with one antibiotic (6.39858 USD). In addition, there were more prescriptions with only one type of antibiotic (*n* = 296; 65.78%), followed by prescriptions with two antibiotics (*n* = 132; 29.33%), and then prescriptions with three antibiotics (*n* = 22; 4.89%).

**Table 5:** Cost analysis of the antibiotic prescriptions

Items				Cost factor ($)			
Number of antibiotics per prescription	Prescriptions with antibiotics	Mean number of drugs per prescription	Sum of items per prescription	Antibiotics cost (total)	Antibiotics cost (mean)	The total cost of items in the prescription (USD)	The average cost of items (USD)
One	296	4.28 ± 1.69	1268	1,482.283	2.66172	3,175.19191	6.39858
Two	132	4.46 ± 1.69	618	2,964.56588	6.57331	1,043.7215	14.0807
Three	22	5.55 ± 1.69	122	139.974	6.36419	237.4882	10.7949

## Discussion

Similar to an earlier report, there were more females prescribed with antibiotics compared with males [4], though this finding varies with some other studies. One, a retrospective study of drug utilization evaluation of antibiotics in *dh uttarakashi* using records of 15 patients, reported male dominance of 53% to 47% females [7]. Second, using a random systematic sampling technique, an observational prospective and prescription-based study conducted in the surgical outpatient department (SOD) and emergency department (ED) in two teaching hospitals, also reported male dominance of 67.9% to 32.1% females [15]. The probable reasons for the more females than males utilizing antibiotics, as seen in our study may be due to the sociological factors such as women being more eager to go to the hospital than men, men and women communicating differently with health professionals, and prescribers having biases that affect their willingness to prescribe antibiotics during consultations with women versus men [16] or that the female population were more exposed to the hazards of infectious diseases [15].

Irrational use of antibiotics is a significant contributor to the development of antimicrobial resistance [17], which has posed a significant threat to the management of infectious diseases. Antibiotics are important category of drugs and its improper use can result in resistance. One of the causes of resistance against antibiotics is the high number of antibiotics prescribed for patients per encounter [4]. The average number of drugs per prescription is an important parameter while doing a prescription audit. Multiple drug prescribing results in poly-pharmacy; this may contribute to irrational prescribing and adverse effects. In this study, the average number of antibiotics per prescription reported was similar to a study conducted among 159 patients visiting Medicine in-patient department of Gauhati Medical College & Hospital in Guwahati (GMCH), Assam, India [18]. Some other studies have reported an average number of antibiotics per the prescription of 1.83, 1.8 and 1.6, respectively, which are slightly higher than our findings [19–21]. In addition, it was found that even though the number of prescriptions with one antibiotic was higher than that with two, and then with three, the average number of drugs per prescription with one, two, and three antibiotics were more than double the average number (2) recommended by the WHO. In other countries, such as Indonesia, Niger, Nigeria, India, Ghana, and Pakistan the prescriptions were made for three or more drugs [22].

The essential medicine list measures the degree to which practices conform to national drug policy, as indicated by prescribing from the national essential medicines list for the type of facility surveyed. As per WHO drug use indicators, out of the total antibiotics prescribed, 379 (84.22%) were from the Nigerian Essential Medicines List. The most common antibiotics prescribed, as we reported, were Amino-penicillins (37.28%) mainly coamoxiclav 625 mg followed by Nitroimidazoles (17.99%) mostly metronidazole, Fluoroquinolones (17.18%) mostly ciprofloxacin, macrolides (13.29%), Cephalosporins (9.24%), Tetracyclines (3.08%), and Lincosamides (0.32%). This is similar to an earlier study that reported highest prescribed antibiotic as Penicillin, followed by Macrolid, Fluoroquinolones, Cephalosporin, and Cephalosporin [4]. Amino-penicillins are bactericidal beta-lactam antibiotics. They are effective against most gram-positive bacteria and are clinically used in treating upper and lower respiratory tract infections, endocarditis urinary tract infection and others. Similarly, an earlier report [4] indicates that most antibiotics prescriptions in the adult outpatient unit are against medical conditions as listed. Essential medicine lists have been shown to improve the quality and cost-effectiveness of health care delivery when combined with proper procurement policies and good prescribing practices. Adequately, in this study, most of the prescriptions were from the essential medicines list.

The percentage of encounter with injections prescribed was far less than the ideal value stipulated by the World Health Organization (13.4–24.1%) were it was found to be 1.8%. Most of the prescriptions were in oral dosage forms. This may be due to patients’ visits to the General out-patient department (GOPD) only at a less severe stage, where extra care is not needed. The less use of injection leads to decreased cost of medication, less tissue necrosis, less anaphylactic shock, reduces chances of transmission of blood-borne diseases, including HIV [23]. An earlier study while assessing the antibiotic prescribing patterns using World Health Organization prescribing indicators at the outpatient Pharmacy Department of University of Gondar referral hospital, Gondar, Northwest Ethiopia, reported that majority of antibiotics were prescribed by oral route (476, 84%) followed by the parenteral route (39, 4%), as observed in this study [4].

## Conclusion

Antimicrobial agents’ utilization study can help in fostering the habits of rational use which means prescribing the right dose, for the right duration, and at the right cost. Thus, urgent steps to increase our knowledge on utilization are needed to identify interventions necessary to promote the rational use of antimicrobials. In this study, though the percentage of drugs prescribed from the essential medicines list was found to be satisfactory, the findings of this study revealed that drug utilization pattern was not optimal following the standard values of WHO prescribing indicators. This study highlights the need to minimize the average number of drugs per prescription and the percentage of antimicrobials prescribed. Though the appropriateness of the antimicrobials prescribed was not evaluated, the need for the introduction of guidelines for prescribing antimicrobials and the role of Hospital Antibiotic Policy must be made mandatory with implementation by the regulatory bodies both at the national and world level. A strict protocol for prescribers is required to promote rational use of antimicrobial agents which would not only prevent resistance but also reduce the treatment expenditure.
